# Combined Exercise Training Performed by Elderly Women Reduces Redox Indexes and Proinflammatory Cytokines Related to Atherogenesis

**DOI:** 10.1155/2019/6469213

**Published:** 2019-08-05

**Authors:** André L. L. Bachi, Marcelo P. Barros, Rodolfo P. Vieira, Gislene A. Rocha, Paula B. M. de Andrade, Angélica B. Victorino, Luiz R. Ramos, Claudia F. Gravina, José D. Lopes, Mauro Vaisberg, Raul C. Maranhão

**Affiliations:** ^1^Department of Otorhinolaryngology, Federal University of São Paulo, São Paulo, Brazil; ^2^Brazilian Institute of Teaching and Research in Pulmonary and Exercise Immunology (IBEPIPE), São Paulo, Brazil; ^3^Interdisciplinary Postgraduate Program in Health Sciences, Institute of Physical Activity Sciences and Sports, Cruzeiro do Sul University, São Paulo, Brazil; ^4^Post-graduation Program in Bioengineering, Brasil University, São Paulo, Brazil; ^5^Post-graduation Program in Sciences of Human Movement and Rehabilitation, Federal University of São Paulo, São Paulo, Brazil; ^6^School of Medicine, Anhembi Morumbi University, São José dos Campos, Brazil; ^7^Department of Neurology and Neurosurgery, Federal University of São Paulo, São Paulo, Brazil; ^8^Department of Preventive Medicine, Federal University of São Paulo, São Paulo, Brazil; ^9^Dante Pazzanese Institute of Cardiology, São Paulo, Brazil; ^10^Department of Microbiology and Immunology, Federal University of São Paulo, São Paulo, Brazil; ^11^Heart Institute, Medical School Hospital, University of São Paulo, São Paulo, Brazil; ^12^Faculty of Pharmaceutical Sciences, University of São Paulo, São Paulo, Brazil

## Abstract

Cardiovascular benefits for the general population of combined aerobic-resistance exercise training are well-known, but the impact of this exercise training modality on the plasma lipid, inflammatory, and antioxidant status in elderly women that are exposed to a great risk of developing ischemic cardio- and cerebrovascular diseases has not been well investigated. So, we aimed to evaluate the plasma lipids, oxidative stress, and inflammatory cytokines in 27 elderly women (TRAINED group, 69.1 ± 8.1 yrs) that were performing moderate intensity combined aerobic-resistance exercise training (3 times/week for at least 18 months) and in 27 sedentary elderly women (SED group, 72.0 ± 6.4 yrs), not submitted to exercise training for at least 5 yrs. Our results showed that BMI was lower in the TRAINED group than in the SED group (25.1 ± 3.2 vs. 28.7 ± 5.1, *p* < 0.05). The TRAINED group had lower glycemia (92 ± 3 vs. 118 ± 12, *p* < 0.05), glycated hemoglobin (5.9 ± 0.1 vs. 6.4 ± 0.2, *p* < 0.05), and triglycerides (98 (75-122) vs. 139 (109-214), *p* < 0.01); equal total cholesterol (199 (175-230) vs. 194 (165-220)), LDL-cholesterol (108 (83-133) vs. 109 (98-136)), and non-HDL-cholesterol (54 (30-74) vs. 62 (26-80)); and also higher HDL-cholesterol (64 (52-77) vs. 52 (44-63), *p* < 0.01) and LDL-C/oxLDL ratio (13378 ± 2570 vs. 11639 ± 3113, *p* < 0.05) compared to the SED group. Proinflammatory cytokines as IL-1*β* (11.31 ± 2.4 vs. 28.01 ± 4.7, *p* < 0.05), IL-6 (26.25 ± 7.4 vs. 49.41 ± 17.8, *p* < 0.05), and TNF-*α* (25.72 ± 2.8 vs. 51.73 ± 4.2, *p* < 0.05) were lower in the TRAINED group than in the SED group. The TRAINED group had lower total peroxides (26.3 ± 7.4 vs. 49.0 ± 17.8, *p* < 0.05) and oxidized LDL (1551 ± 50.33 vs. 1773 ± 74, *p* < 0.02) and higher total antioxidant capacity (26.25 ± 7.4 vs. 49.41 ± 17.8, *p* < 0.001) compared to the SED group. In conclusion, in TRAINED women, BMI was lower, plasma lipid profile was better, plasma oxidative stress was diminished, and there was less expression of proinflammatory interleukins than in SED, suggesting that combined aerobic-resistance exercise training may promote the protection against the complications of ischemic cardio- and cerebrovascular disease in elderly women.

## 1. Introduction

The pace of population aging is being accelerated, and the WHO estimated that between 2015 and 2050 the proportion of the world population over 60 years will grow from 12% to 22% [1]. Thus, the widespread promotion of healthy aging is mandatory, inclusively, to keep the pace with the spending of the health systems.

During the aging process, it is common to observe increases of adipose tissue in the viscera and organs such as the liver and muscles [2]. Overweight and obesity are clearly associated with alterations in the lipid profile and raise in the markers of systemic inflammation, including C-reactive protein (CRP) and proinflammatory cytokines, such as interleukin- (IL-) 1 beta (IL-1*β*), IL-6, and tumor necrosis factor alpha (TNF-*α*) [2, 3]. In the elderly, the increase in the systemic markers of inflammation has been pointed as risk factors for chronic diseases such as atherosclerosis, cancer, sarcopenic syndromes, and diabetes mellitus, among others [4].

Alterations in the plasma metabolism and concentration of lipoproteins [5], redox imbalance, and dysregulation of the inflammatory response [6] in the vessel are key factors in the development, progression, and clinical manifestations of atherosclerosis. In fact, lipid peroxidation begins with the previous accumulation of lipid peroxides (LOOH). Redox decomposition of LOOH molecules initiates chain reactions promoted by alkyl, peroxyl, and alkoxyl radicals (L^·^, LOO^·^, and LO^·^, respectively) that further oxidize other lipid and protein molecules through massive production of reactive carbonyl species [7]. Moreover, bioactive “oxylipids” or aldehyde derivatives are involved in the activation of immune responses and accumulation of neutrophil/macrophage in atherosclerotic lesions culminating in an aggravated vascular condition [8].

The beneficial effects in the elderly population of aerobic and of resistance exercise training on the plasma lipid metabolism, oxidative, and inflammatory processes have been documented in the literature [9]. Whereas the aerobic training preferentially improves cardiovascular fitness, resistance training increases the muscle mass and both training modalities promote loss of body fat mass [9, 10]. It is widely accepted that the regular practice of exercise training, both aerobic (endurance) and resistance (anaerobic/strength) sets, is one of the most effective nonpharmacological interventions that can partially reverse the effects of vascular dysfunction, thereby decreasing the risk of death and consequently increase longevity [11–13]. Although it has been generally agreed that combined aerobic-resistance exercise training may equally attain both cardiovascular and muscle targets [14], the effects of combined aerobic-resistance exercise training on the lipid profile, oxidative stress, and inflammatory markers of atherosclerosis have been scarcely explored in aged subjects. These data are particularly important in women because the female life expectancy is longer than that of men. Those considerations lead us to investigate the status of plasma lipids and oxidized LDL (oxLDL), redox indexes, and inflammatory markers in elderly women under a combined aerobic-resistance exercise training program as compared to sedentary women in the equivalent age range.

## 2. Methods

### 2.1. Study Subjects

**Figure 1:**
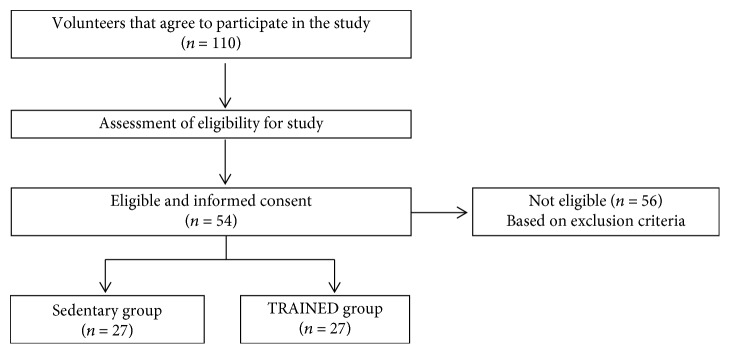
Flow diagram of the study.

Fifty-four volunteer elderly women, aged 60-80 years, were selected for the study. A flow diagram is shown in Figure 1. Twenty-seven were participating in a combined aerobic-resistance exercise program (TRAINED group) sponsored by the municipality of the city of São Paulo, and twenty-seven age-paired women were sedentary (SED group) participants of a primary health care program of the Department of Preventive Medicine of the Federal University of São Paulo Medical School. Both groups of women were residents in the same neighborhood.

None of the subjects presented asthma, type-1 diabetes mellitus; neoplastic, renal, or liver diseases; dementia; thrombosis; or manifested cardiovascular disease. None was under statin or other lipid-lowering drugs. The participants responded to a Food Frequency Questionnaire in which the consumption of antioxidants was assessed. The study was in agreement with the Declaration of Helsinki and with the Ethical Standards defined by Harriss and Atkinson [15] and was approved by the Ethics Committee of the Federal University of São Paulo (UNIFESP, protocol number 0788/10). The volunteers signed an informed written consent form.

### 2.2. Combined Aerobic-Resistance Exercise Training Program

The physical exercise protocol is a combination of aerobic and resistance exercises performed in moderate intensity (Table 1). The combined aerobic-resistance exercise training followed the guidelines for exercise prescription recommended by the American College of Sports Medicine [9, 16].

Volunteers from the TRAINED group performed their prescribed exercises during 60-75 minutes per session, 3 times a week, on nonconsecutive days, for at least 18 months. The same experienced instructor supervised all the volunteers.

A description of combined physical exercise regime performed by the TRAINED group is described in Table 1.

Volunteers of the SED group, although independent and active, were not involved in any regular exercise program for at least five years, and they were oriented to maintain their normal routine during the study.

### 2.3. Sample Collection

Blood sampling occurred at 8:00 AM after a 12 h fast. The TRAINED group performed its last exercise training session at least 24 h beforehand. Serum/plasma aliquots of 500 *μ*L were obtained after centrifugation (10 min, 400xg) of blood samples and stored at -80°C.

### 2.4. Laboratorial Analysis

Plasma total cholesterol concentration was measured by the CHOD-PAP method using commercial kits (Kovalente, São Gonçalo, Brazil), and the results were analyzed with an automated system (Dimension® RxL Max® Integrated Chemistry System, Siemens, Deerfield, IL, USA). HDL-C and triglyceride concentrations were determined using commercial kits and an automated analysis system (ADVIA® 2400, Siemens, Deerfield, IL, USA). LDL-C was estimated by the Friedewald formula [17]. Plasma glucose concentration and glycated hemoglobin percentage were measured by commercial kits and an automated analysis system (ADVIA® 2400, Siemens, Deerfield, IL, USA).

Serum concentration of oxLDL was evaluated by ELISA, using the Indirect Enzyme Immunoassay kit (USCN® Life Science Inc., Wuhan, China), total antioxidant capacity (TAC) by a colorimetric commercial kit (Cayman Chemical Corporation, Ann Arbor, MI, USA), total lipid peroxide content (LOOH) using the QuantiChrom™ Peroxide Assay Kit, a colorimetric commercial kit (BioAssay Systems, Hayward, CA, USA), and proinflammatory cytokines (IL-1*β*, IL-6, and TNF-*α*) using Millipore® Multiplex Assays Using Luminex® (EMD Millipore Corporation, Billerica, MA, USA). All analyses were performed in accordance with the manufacturer's instructions.

### 2.5. Statistical Analysis

All data were previously analyzed by the Shapiro-Wilk test used to evaluate the normal distribution and after by the Levene test used to evaluate the variance homogeneity. Student's *t*-test was used to analyze the differences in age, height, weight, and body mass index (BMI). The Mann-Whitney test was used to determine the differences in plasma lipids and glucose, glycated hemoglobin, oxLDL, total peroxides, TAC, proinflammatory cytokines (IL-1*β*, IL-6, and TNF-*α*), and the relation between plasma concentrations of LDL-C and oxLDL ((LDL − C/oxLDL) × 100). Spearman's rank correlation coefficient was employed to identify any correlation between oxLDL and BMI or LDL-C or TAC or total peroxides. The significance level was set to 5% (*p* < 0.05).

## 3. Results

Physical and clinical characteristics of the volunteers are presented in Table 2. No differences were observed between the two groups regarding the antioxidant or vitamin intake of the participant subjects.

As shown in Table 2, the TRAINED group had lower BMI than the SED group (*p* = 0.002).  Table 3 shows that both plasma glucose (*p* = 0.02) and glycated hemoglobin (*p* = 0.04) were lower in the TRAINED group than in the SED group. LDL-C and non-HDL-C were similar but HDL-C was higher (*p* = 0.001) and triglycerides were lower (*p* = 0.008) in the TRAINED group compared to the SED group.

**Figure 2:**
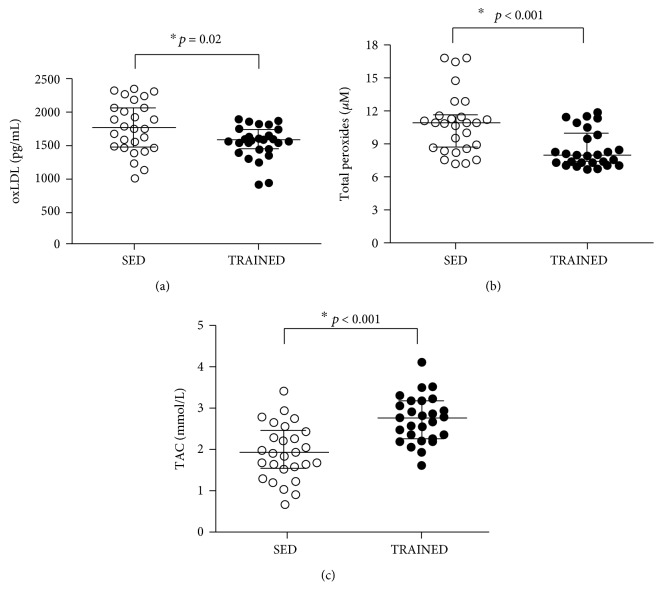
Serum concentrations of oxidized LDL (oxLDL, pg/mL (a)), lipid peroxide (LOOH, *μ*M (b)), and plasma total antioxidant capacity (TAC, mmol/L (c)) in the SED and TRAINED groups were statistically analyzed using the Mann-Whitney test. These data were presented as the median (interquartile range) with a significance level of ^∗^*p* < 0.05.

As shown in Figure 2, the TAC serum concentration was higher (*p* < 0.001), whereas LOOH (*p* < 0.001) and oxLDL (*p* = 0.02) were lower in the TRAINED group than in the SED group. In addition, as shown in Table 3, the relationship between LDL-C and oxLDL ((LDL − C/oxLDL) × 100) was higher in the TRAINED group than in the SED group (*p* = 0.04).

**Figure 3:**
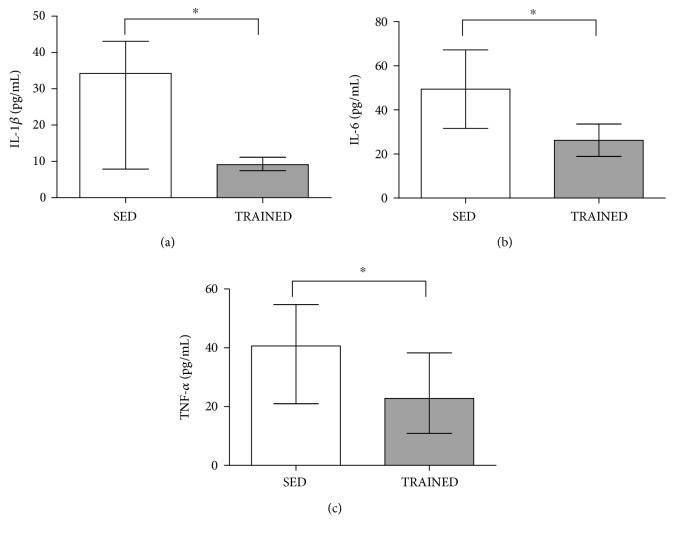
Serum concentrations (pg/mL) of IL-1*β* (a), IL-6 (b), and TNF-*α* (c) in the SED and TRAINED groups were statistically analyzed using the Mann-Whitney test. These data were presented as the median (interquartile range) with a significance level of ^∗^*p* < 0.05.

Figure 3 shows that the serum concentration of proinflammatory cytokines IL-1*β* (*p* = 0.001), IL-6 (*p* = 0.02), and TNF-*α* (*p* = 0.01) was higher in the SED group than in the TRAINED group.

**Figure 4:**
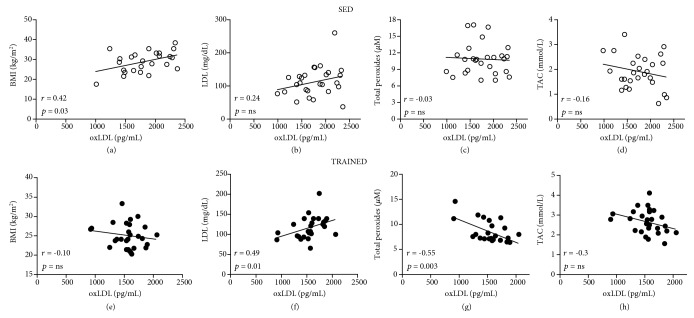
Spearman's rank correlation coefficient analysis was used to identify the correlation between oxidized LDL (oxLDL, pg/mL) and BMI (kg/m^2^ (a, e)) or LDL-C (mg/dL (b, f)) or lipid peroxide (LOOH, *μ*M (c, g)) or total antioxidant capacity (TAC, mmol/L (d, h)) in the SED group (a–d) and the TRAINED group (e–h). Significance level of ^∗^*p* < 0.05.

Spearman's coefficient analysis (Figure 4) showed a positive correlation between BMI and oxLDL concentration in the SED group (Figure 4(a)), which was not observed in the TRAINED group (Figure 4(e)). LDL-C and oxLDL were linearly correlated in the TRAINED group. However, this correlation was not significant in the SED group (Figure 4(f)). Moreover, LOOH values were correlated with those of oxLDL in the TRAINED group (Figure 4(g)). No other significant correlations were found.

## 4. Discussion

In this transversal study enrolling elderly women, it was shown that the regular practice of combined aerobic-resistance exercise leads to a clear-cut improvement of markers of lipid and inflammatory status and of the oxidative stress that are related to the prevention of the manifestations of atherosclerosis.

LDL-C was not lower in the TRAINED group than in the SED group, which is in accordance with studies showing that several modalities of exercise training do not alter LDL-C plasma levels [10, 18]. However, regardless of the lack of effect on LDL-C levels, exercise training can improve the LDL metabolism by increasing the LDL removal from the plasma and the lipoprotein turnover, as shown in the study by Vinagre et al. [19]. In that study, the plasma kinetics of LDL-like particles measured in cycling practitioners was shown to increase severalfold in comparison with sedentary subjects, possibly due to an exercise-training induction of LDL receptor overexpression. In addition, the shorter residence time of LDL in the bloodstream can conceivably decrease the exposure of the lipoprotein to oxidation, and in fact, those authors found a correlation between the LDL-like particle clearance and the plasma levels of oxLDL [19]. Resistance exercise training program administered to healthy sedentary men had also the ability to increase LDL clearance with lowering of the oxLDL levels [20]. Sedentary hypercholesterolemic subjects had the LDL clearance increased after an aerobic exercise training program, with a concomitant decrease of the LDL susceptibility to oxidation [21]. In the current study, the lower oxLDL levels achieved by combined aerobic-resistance exercise training can presumptively be ascribed to increased LDL clearance, in view of the above-mentioned previous studies.

Besides the decrease in the plasma levels of oxLDL, an established risk factor for ischemic cardio- and cerebrovascular diseases [22–26], we also documented a decrease in the plasma LOOH levels in the TRAINED group. Higher LOOH plasma levels are also a risk factor for ischemic cardio- and cerebrovascular diseases [27–30]. According to Wonisch et al. [31], the oxidative stress increases with increasing BMI and age and, as a consequence, occurs an increase of peroxide molecules that can lead to an unbalanced lipid profile. So, the BMI values found in the TRAINED group and also the negative correlation found in our study between LOOH and the oxLDL levels reinforce the consistence of the favorable effects of the combined aerobic-resistance exercise training on the redox indexes. Moreover, we also found that in the TRAINED group, TAC levels were higher than in the SED group, which could buffer oxidative stress, thereby preventing the LDL oxidation [32]. Previous studies have also shown beneficial actions of exercise training protocols, including combined aerobic-resistance exercise training on the antioxidant defenses [33–35]. Taken together, these results support the assumption that combined aerobic-resistance exercise training can be a valid tool to improve the body antioxidation mechanisms and thereby atherosclerosis prevention.

Here, the TRAINED group exhibited higher levels of HDL-C as compared to the SED group. Exercise training is a classical factor to promote an increase in HDL-C [9, 10, 36–38], and some studies had already reported that combined aerobic-resistance exercise training also has the capability of HDL-C increase [10, 18]. It is noteworthy that the effects on HDL-C are largely related to exercise training intensity [9, 10]. HDL-C levels are inversely correlated with the risk of ischemic cardio- and cerebrovascular diseases [39, 40]. However, this lipoprotein has several functions that are protective against atherosclerosis and other diseases that are not necessarily dependent of the HDL-C levels. Among these functions, HDL is the main lipoprotein to exert cholesterol esterification and reverse cholesterol transport and has also many other protective functions such as antioxidant, promoter of vasodilation, antiapoptotic, and anti-inflammatory [40]. Previously, we have shown that combined aerobic-resistance exercise training performed in aged women increased the transfer of cholesterol to HDL [41]. This effect is presumably beneficial, since patients with cardiovascular disease exhibit low cholesterol transfer values compared to controls without the disease [42, 43].

As judged from the comparison of the TRAINED and SED groups, the practice of combined aerobic-resistance exercise training also had anti-inflammatory actions, since the TRAINED group presented lower serum levels of proinflammatory cytokines than the SED group. Similar to our results, some authors have reported that combined aerobic-resistance exercise training improves the inflammatory status [44–46]. It is reasonable to hypothesize that these anti-inflammatory effects may be associated with the antioxidant effects of exercise training observed here, since the relationship between chronic inflammatory processes and oxidative stress, especially in aging, is well-known [47, 48].

It is also widely accepted that exercise training is a chief factor to attain the control of glycemic levels, which was also documented here and in other studies [9, 49, 50].

As a limitation of this study, it should be mentioned that a longitudinal interventional study would be rigorously more appropriate to establish the biohumoral changes elicited by exercise training. However, in this cross-sectional protocol, the results of long-standing (over 18 months) regular practice of training, as compared to nonpractitioners of the same community, offer a fair evaluation of individuals in their real-life conditions.

## 5. Conclusion

The results of the current cross-sectional study suggest that elderly women may benefit from the regular practice of combined aerobic-resistance exercise training in many metabolic aspects that are related to protection against the complications of ischemic cardio- and cerebrovascular disease.

## Figures and Tables

**Table 1:** Description of combined physical exercise regime performed by the TRAINED group.

Aerobics	Exercise frequency: 60 to 70% of the maximal heart rate reserve (MHHR), calculated by the equation (208‐0.7 × age) proposed by Tanaka et al. [51]
	Exercise type: physical exercises in step platform, jump, coordination, and rhythmic movements (dance sometimes)
	Impact: low impact
	Monthly cardiac control: Polar, FT1, Finland

Resistance	At least 5 different exercises for different muscle groups (upper and lower limb muscles, abdomen, gluteus, and muscles related to core/postural stabilization, including dorsal and lumbar muscles)
	Performed slowly in two series with 10-20 repetitions, between 50 and 60% of 1 RM (repetition maximum)
	Different combinations of two muscle groups (described above) performed in four consecutive sessions
	Borg scale to adjust the weight load monthly

**Table 2:** Physical (means ± SD) and clinical characteristics of the TRAINED and SED groups. Significance level of ^∗^*p* < 0.05.

Characteristics	Volunteers (*n* = 54)		
	SED (*n* = 27)	TRAINED (*n* = 27)	*p* value
Physical			
Age (year)	72.0 ± 6.4	69.1 ± 8.1	0.257
Height (m)	1.55 ± 0.05	1.54 ± 0.07	0.543
Weight (kg)	71.2 ± 13.3^∗^	59.8 ± 9.3	0.005
Body mass index (kg/m^2^)	28.7 ± 5.1^∗^	25.1 ± 3.2	0.002
Clinical (*n*)^#^			
Type 2 diabetes mellitus	6	4	>0.05
Dyslipidemia	13	12	>0.05
Obesity	4	3	>0.05
Arterial hypertension	15	15	>0.05
Depression	2	1	>0.05
Lifestyle			
Physical exercise training			
≤2 times/week	0	0	>0.05
≥2 times/week	0^∗^	27	0.0001
Current alcohol use	0	0	
Smoking			
Current smoker	1	1	>0.05

^#^ *n* = number of individuals.

**Table 3:** Values of the lipid profile and glucose are expressed as the median and interquartile range (mg/dL); glycated hemoglobin (HbA1c) is expressed in percentage (%) and oxLDL is expressed in means ± SD (pg/mL) in the TRAINED and SED groups. Significance level of ^∗^*p* < 0.05.

Variables	Volunteers (*n* = 54)		
	SED (*n* = 27)	TRAINED (*n* = 27)	*p* value
Glucose (mg/dL)	117.8 ± 11.6^∗^	91.9 ± 2.5	0.02
HbA1c (%)	6.4 ± 0.2^∗^	5.9 ± 0.1	0.04
Cholesterol (mg/dL)			
Total	194 (165-220)	199 (175-230)	0.709
LDL	108 (83-133)	109 (98-136)	0.721
Non-HDL	62 (26-80)	54 (30-74)	0.467
HDL	52 (44-63)^∗^	64 (52-77)	0.001
Triglycerides (mg/dL)	139 (109-214)^∗^	98 (75-122)	0.008
oxLDL (pg/mL)	1773 ± 384.8^∗^	1551 ± 261.5	0.02
LDL/oxLDL ratio	11639 ± 3113^∗^	13378 ± 2570	0.04

## Data Availability

The data used to support the findings of this study are included within the article.
